# COVID-19 Outbreak Associated with a SARS-CoV-2 R.1 Lineage Variant in a Skilled Nursing Facility After Vaccination Program — Kentucky, March 2021

**DOI:** 10.15585/mmwr.mm7017e2

**Published:** 2021-04-30

**Authors:** Alyson M. Cavanaugh, Sarah Fortier, Patricia Lewis, Vaneet Arora, Matt Johnson, Karim George, Joshua Tobias, Stephanie Lunn, Taylor Miller, Douglas Thoroughman, Kevin B. Spicer

**Affiliations:** ^1^Epidemic Intelligence Service, CDC; ^2^Kentucky Department for Public Health; ^3^Center for Preparedness and Response, CDC; ^4^Division of Healthcare Quality Promotion, National Center for Emerging and Zoonotic Infectious Diseases, CDC.

Although COVID-19 mRNA vaccines demonstrated high efficacy in clinical trials (*1*), they were not 100% efficacious. Thus, some infections postvaccination are expected. Limited data are available on effectiveness in skilled nursing facilities (SNFs) and against emerging variants. The Kentucky Department for Public Health (KDPH) and a local health department investigated a COVID-19 outbreak in a SNF that occurred after all residents and health care personnel (HCP) had been offered vaccination. Among 83 residents and 116 HCP, 75 (90.4%) and 61 (52.6%), respectively, received 2 vaccine doses. Twenty-six residents and 20 HCP received positive test results for SARS-CoV-2, the virus that causes COVID-19, including 18 residents and four HCP who had received their second vaccine dose >14 days before the outbreak began. An R.1 lineage variant was detected with whole genome sequencing (WGS). Although the R.1 variant has multiple spike protein mutations, vaccinated residents and HCP were 87% less likely to have symptomatic COVID-19 compared with those who were unvaccinated. Vaccination of SNF populations, including HCP, is critical to reduce the risk for SARS-CoV-2 introduction, transmission, and severe outcomes in SNFs. An ongoing focus on infection prevention and control practices is also essential.

## Investigation and Epidemiologic Findings

The SNF conducted vaccination clinics using Pfizer-BioNTech mRNA vaccine on January 10, January 31, and February 21, 2021. Among 83 residents and 116 HCP, 75 (90.4%) and 61 (52.6%), respectively, received two vaccine doses. All vaccinated residents and HCP were vaccinated on-site, the majority on January 10 and 31. Four residents and five HCP received their second dose during the third clinic, which was <14 days before the outbreak onset.

Before and during the outbreak, SARS-CoV-2 testing was used for evaluating symptomatic illness in residents and HCP. Symptom screening of residents and HCP had been ongoing since March 2020, and twice-weekly screening testing of all HCP had been occurring since November 2020. A COVID-19 case was defined as a positive SARS-CoV-2 antigen or reverse transcription–polymerase chain reaction (RT-PCR) test result. Possible reinfection was defined as a positive SARS-CoV-2 test result >90 days after a previous laboratory-confirmed infection.

The outbreak was identified during routine HCP antigen testing on March 1.* This was 8 days after the third vaccination clinic. The index case occurred in an unvaccinated, symptomatic HCP. Once the outbreak was identified, daily rapid point-of-care antigen testing of all residents, regardless of symptoms, was added to the twice-weekly HCP testing. Additional specimens were collected the same day for RT-PCR confirmation of positive antigen test results. One week after the outbreak was identified, resident antigen testing was reduced to three times weekly, then to twice weekly after no additional cases were identified for 1 week.

The local health department interviewed HCP and facility staff members to collect information about the cases. Vaccination status was ascertained through immunization registry review and facility interviews. COVID-19–related hospitalizations and deaths were confirmed by medical records reviews. This activity was reviewed by CDC and was conducted consistent with applicable federal law and CDC policy.^†^

Relative risks (RRs) were calculated comparing unvaccinated and vaccinated residents and HCP; vaccine effectiveness (VE [1−RR of vaccinated versus unvaccinated x 100]) was calculated for the following outcomes: SARS-CoV-2 infection, symptomatic COVID-19, hospitalization, and death. Persons who received their second vaccine dose ≥14 days before the outbreak began were considered vaccinated, consistent with CDC postvaccination guidance^§^ and breakthrough case definition. Ten persons who had received at least 1 dose but had not received a second vaccine dose ≥14 days before the outbreak were excluded from analyses.

A sensitivity analysis was conducted using a 7-day threshold to classify persons as vaccinated, consistent with the Pfizer-BioNTech vaccine clinical trials (*1*). Four residents and five HCP who received their second vaccine dose 8 days before outbreak identification were classified as vaccinated in this sensitivity analysis. One HCP who received a single vaccine dose remained excluded (Supplementary Table https://stacks.cdc.gov/view/cdc/105235).

KDPH Division of Laboratory Services performed WGS (*2*). Genomes were assembled using the StaPH-B Monroe pipeline,^¶^ followed by Nextclade** for clade assignment and mutation calling, Pangolin^††^ for lineage assignment, and Nextstrain for phylogenetic analysis (*3*).

During the outbreak, 46 COVID-19 cases were identified, including cases in 26 residents (18 fully vaccinated) and 20 HCP (four vaccinated) (Figure) (Table). Two cases occurred in residents who had received their second vaccine dose within 14 days; these two cases were excluded from the primary analysis. Vaccinated residents and HCP were less likely to be infected than were unvaccinated persons. Attack rates in unvaccinated residents (75.0%) were 3 times as high as those in vaccinated residents (25.4%; RR = 3.0; 95% confidence interval [CI] = 1.7–5.2) and in unvaccinated HCP (29.6%) were 4.1 times as high as those in vaccinated HCP (7.1%; RR = 4.1; 95% CI = 1.5–11.6). The estimated VE against SARS-CoV-2 infection among residents was 66.2% (95% CI = 40.5%–80.8%) and among HCP was 75.9% (95% CI = 32.5%–91.4%).

**FIGURE:**
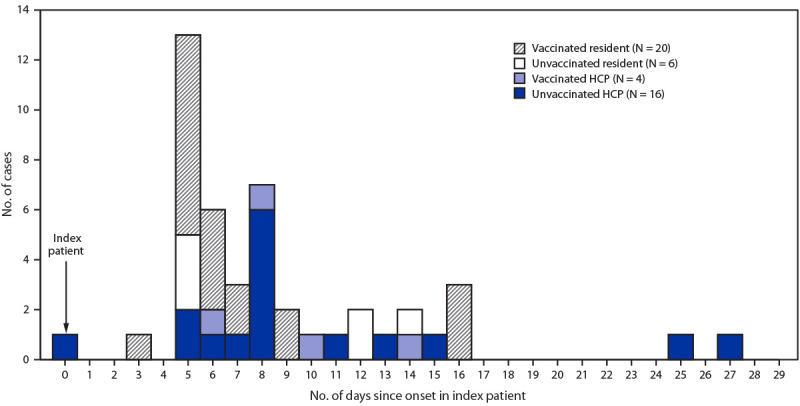
SARS-CoV-2 illness onset* among residents and health care personnel (HCP) in a skilled nursing facility, relative to onset in the index patient, by vaccination status^†^ — Kentucky, March 2021 * Symptom onset date or specimen collection date, if asymptomatic. ^†^ Persons who received 2 doses of Pfizer-BioNTech vaccine are indicated as vaccinated; unvaccinated persons received no vaccine doses. Persons who received a second dose of vaccine <14 days before outbreak onset (four residents and five HCP) and those who received only a single dose of vaccine (one HCP) were excluded from the primary analysis; this resulted in exclusion of two cases that occurred in residents.

**TABLE:** Relative risk and estimated vaccine effectiveness for prevention of SARS-CoV-2 infection, symptomatic COVID-19, hospitalization, and death for fully vaccinated persons compared with unvaccinated persons during a COVID-19 outbreak in a skilled nursing facility (SNF) — Kentucky, 2021

Population and outcome	No. (% attack rate)		Unvaccinated versus vaccinated RR (95% CI)	Vaccine effectiveness^§^ (95% CI)
	Vaccinated*	Unvaccinated^†^		
**Total SNF population^¶^**	**(n = 127)**	**(n = 62)**	—	—
SARS-CoV-2 infection	22 (17.3)	22 (35.5)	—	—
Symptomatic	8 (6.3)	20 (32.3)	—	—
Hospitalization	2 (1.6)	4 (6.5)	—	—
Death	1 (0.8)	2 (3.2)	—	—
**Residents**	**(n = 71)**	**(n = 8)**	—	—
SARS-CoV-2 infection	18 (25.4)	6 (75.0)	3.0 (1.7–5.2)	66.2 (40.5–80.8)
Symptomatic	6 (8.5)	5 (62.5)	7.4 (2.9–18.8)	86.5 (65.6–94.7)
Hospitalization	2 (2.8)	4 (50.0)	17.8 (3.8–82.1)	94.4 (73.9–98.8)
Death	1 (1.4)	2 (25.0)	17.8 (1.8–174.7)	94.4 (44.6–99.4)
**Health care personnel**	**(n = 56)**	**(n = 54)**	—	—
SARS-CoV-2 infection	4 (7.1)	16 (29.6)	4.1 (1.5–11.6)	75.9 (32.5–91.4)
Symptomatic	2 (3.6)	15 (27.8)	7.8 (1.9–32.4)	87.1 (46.4–96.9)
Hospitalization	0 (—)	0 (—)	—	—
Death	0 (—)	0 (—)	—	—

**Abbreviations:** CI = confidence interval; RR = relative risk.

* Receipt of 2 doses of Pfizer-BioNTech vaccine ≥14 days before identification of the SNF outbreak; persons who received a second dose of vaccine <14 days before the outbreak (four residents and five health care personnel) and those who received a single dose of vaccine (one health care worker) were excluded, which resulted in exclusion of two resident cases.

^†^ Receipt of zero doses of COVID-19 vaccine.

^§^ Calculated as (1−RR of vaccinated versus unvaccinated) x100.

**^¶^** Includes residents and health care personnel.

VE against symptomatic COVID-19 was 86.5% (95% CI = 65.6%–94.7%) among residents and 87.1% (95% CI = 46.4%–96.9%) among HCP. VE against hospitalization was 94.4% (95% CI = 73.9%–98.8%) among residents; no HCP were hospitalized. Three residents died, two of whom were unvaccinated (VE = 94.4%; 95% CI = 44.6%–99.4%).

Four possible reinfections were identified (one resident and three HCP); of these, one HCP was vaccinated. All four persons experienced symptomatic illness. One resident was infected 300 days earlier and had nine consecutive negative RT-PCR tests before reinfection, including two within 30 days of the outbreak. This resident was hospitalized and died.

## Laboratory and Bioinformatics Findings

WGS was performed for 28 specimens (27 persons, including one who was reinfected); all had >97% genome coverage at a depth of >30x, therefore passing required quality control matrices.^§§^ Examination of phylogeny revealed 28 clustered sequences sharing 14 amino acid mutations not present in the reference Wuhan-1 genome: ORF1a:A2584T, ORF1b:P314L, ORF1b:G1362R, ORF1b:P1936H, S:E484K, S:D614G, S:G769V, S:W152L, M:F28L, N:M1X, N:S187L, N:R203K, N:G204R, and N:Q418H. This cluster aligns with the R.1 lineage, which had not previously been identified in Kentucky. Whereas the 28 sequences share spike protein mutations E484K, D614G, G769V, and W152L with the R.1 root, the mutation ORF1a:A2584T places the cluster in a separate group on the phylogenetic tree.

## Public Health Response

The local health department, along with the KDPH regional epidemiologist and regional infection preventionist, provided guidance on implementation of infection prevention strategies. These included the use of transmission-based precautions and hand hygiene, ongoing testing to identify new cases, exclusion of symptomatic HCP from work, isolation and quarantine of HCP, and provision of dedicated and separate spaces for care of infected and exposed residents, regardless of vaccination status.^¶¶^

## Discussion

In a SNF with 90.4% of residents vaccinated, an outbreak of COVID-19 occurred after introduction from an unvaccinated, symptomatic HCP. WGS identified an R.1 lineage variant, characterized by E484K and other mutations within the spike protein. Attack rates were three to four times as high among unvaccinated residents and HCP as among those who were vaccinated; vaccinated persons were significantly less likely to experience symptoms or require hospitalization.

Although the R.1 variant is not currently identified as a CDC variant of concern or interest,*** it does have several mutations of importance. The D614G mutation demonstrates evidence of increasing virus transmissibility (*4*). The E484K mutation, found within the receptor-binding domain of the spike protein, is also seen in the variants of concern B.1.351 and P.1, which show evidence of reduced neutralization by convalescent and postvaccination sera (*5*,*6*). Mutation W152L might reduce the effectiveness of neutralizing antibodies (*7*). Although vaccination was associated with decreased likelihood of infection and symptomatic illness, 25.4% of vaccinated residents and 7.1% of vaccinated HCP were infected, supporting concerns about potential reduced protective immunity to R.1. In addition, four possible reinfections were identified, providing some evidence of limited or waning natural immunity to this variant.

Point estimates for VE against SARS-CoV-2 infections were lower than were those reported from Israel’s national vaccination program (*8*). Whereas this could reflect reduced protection against R.1, other factors to consider include the smaller sample size in this study and the higher exposure risk associated with an outbreak in a congregate setting. In addition, testing, regardless of symptoms, was performed with high frequency for both residents and HCP, which contrasts with VE studies that use a primary reliance on individual test-seeking behavior. Such differences could influence VE estimates for infection; therefore, caution is urged when comparing these studies. Regardless of VE differences in SARS-CoV-2 infection, the estimated VE for COVID-19 symptom prevention (86.5% for residents; 87.1% for HCP) demonstrates a strong protective effect of vaccination.

The risk for poor outcomes among unvaccinated SNF residents is highlighted by the hospitalization of four of the six unvaccinated, infected residents, and two subsequent deaths, including in one previously infected resident. This underscores the importance of the Advisory Committee on Immunization Practices’ recommendation that all persons, including those who have recovered from COVID-19, be vaccinated.^†††^

Low acceptance of vaccination among SNF HCP might increase the likelihood of SARS-CoV-2 introduction and transmission within a facility. Nationally, a median of 37.5% of HCP working in long-term care facilities had received at least 1 dose of vaccine by mid-January 2021 (*9*). Although the vaccination rate in this SNF surpassed this early national rate, approximately one half of HCP were vaccinated. To protect SNF residents, it is imperative that HCP, as well as SNF residents, be vaccinated. A continued emphasis on strategies for prevention of disease transmission, even among vaccinated populations, is also critical. Timely implementation of infection control strategies after outbreak identification likely contributed to the rapid decline in new cases during the second week of the outbreak.

The findings in this report are subject to at least three limitations. First, the health status of residents who declined vaccination might have differed from those who consented to vaccination. Thus, hospitalization and death outcomes might be biased when comparing the groups without controlling for underlying health conditions. Second, underlying health status and advance directives might affect decisions for resident hospitalization; therefore, association of vaccination with hospitalization in this SNF population might have limited generalizability. Finally, because of the reduced sensitivity of antigen testing in asymptomatic populations,^§§§^ it is possible that some asymptomatic cases were not identified. If this introduced differential bias for identification of cases in either the vaccinated or unvaccinated groups, actual VE for the prevention of SARS-CoV-2 infections could differ from measured effectiveness.

An R.1 lineage variant, not previously detected in Kentucky, was identified in a SNF outbreak where 46 residents and HCP were infected. Compared with unvaccinated persons, vaccinated persons had reduced risk for SARS-CoV-2 infection and symptomatic COVID-19. A continued emphasis on vaccination of SNF populations, including HCP, is essential to reduce the risk for SARS-CoV-2 introduction, transmission, and severe outcomes in SNFs. An ongoing focus on infection prevention and control practices is also critical.

**Summary**

**What is already known about this topic?**

COVID-19 vaccines have demonstrated high efficacy in clinical trials. Limited data are available on effectiveness in skilled nursing facilities (SNFs) and against emerging variants.

**What is added by this report?**

In a COVID-19 outbreak at a Kentucky SNF involving a newly introduced variant to the region, unvaccinated residents and health care personnel (HCP) had 3.0 and 4.1 times the risk of infection as did vaccinated residents and HCP. Vaccine was 86.5% protective against symptomatic illness among residents and 87.1% protective among HCP.

**What are the implications for public health practice?**

Vaccination of SNF residents and HCP is essential to reduce the risk for symptomatic COVID-19, as is continued focus on infection prevention and control practices.

## Footnotes

*
https://www.cdc.gov/coronavirus/2019-ncov/hcp/nursing-homes-testing.html

† 45 C.F.R. part 46.102(l)(2), 21 C.F.R. part 56; 42 U.S.C. 241(d); 5 U.S.C. 552a; 44 U.S.C. 3501 et seq.

§
https://www.cdc.gov/coronavirus/2019-ncov/vaccines/fully-vaccinated-guidance.html

¶
https://staph-b.github.io/staphb_toolkit/workflow_docs/monroe/

**
https://clades.nextstrain.org/

††
https://github.com/cov-lineages/pangolin

§§ Percentage of coverage and depth of coverage are metrics computed during genome assembly that correspond with data quality. The data quality threshold used by the Association of Public Health Laboratories is ≥90% and 10X depth for Illumina sequencing technology. https://www.aphl.org/programs/preparedness/Crisis-Management/Documents/APHL-SARS-CoV-2-Sequencing.pdf

¶¶
https://www.cdc.gov/coronavirus/2019-ncov/hcp/long-term-care.html

***
https://www.cdc.gov/coronavirus/2019-ncov/cases-updates/variant-surveillance/variant-info.html

†††
https://www.cdc.gov/vaccines/covid-19/info-by-product/clinical-considerations.html

§§§
https://www.cdc.gov/coronavirus/2019-ncov/lab/resources/antigen-tests-guidelines.html
